# The Influence of Group Competence on Individual Willingness to Join

**DOI:** 10.3390/bs16050821

**Published:** 2026-05-19

**Authors:** Xiangwei Kong, Bin Zuo, Yatian Lei, Fangfang Wen

**Affiliations:** 1 School of Psychology, Central China Normal University, Wuhan 430079, China; xwkong@mails.ccnu.edu.cn; 2 Department of Psychology, Sun Yat-Sen University, Guangzhou 510275, China; 3 Department of Psychology, School of Education Science, Hunan Normal University, Changsha 410081, China; 4 Institute of Interdisciplinary Studies, Hunan Normal University, Changsha 410081, China

**Keywords:** group competence, group affiliation decisions, individual competence, group identification, individual willingness to join

## Abstract

In everyday life, individuals frequently encounter situations in which they may join new groups; however, previous research has primarily focused on issues that arise after group formation, leaving insufficient attention to the processes preceding spontaneous group affiliation. This study investigates how different levels of group competence influence individuals’ willingness to join, using Optimal Distinctiveness Theory as its theoretical framework. Through two experiments, it systematically examines participants’ willingness to join groups of varying competence levels and how this willingness is moderated by participants’ own competence. The results indicate that high-competence groups demonstrate stronger member attractiveness and effectively promote individuals’ willingness to join. Crucially, the group’s attraction to individuals is moderated by individuals’ own competence. When group competence is lower than one’s own competence level, willingness to join increases as group competence rises toward one’s own level. Strikingly, when group competence exceeds one’s own, willingness to join remains uniformly high and stable, rather than decreasing as Optimal Distinctiveness Theory would predict. These findings suggest that individuals engage in a psychological trade-off based on competence alignment when autonomously choosing whether to join social or professional groups. We interpret this pattern as evidence for a “downward aversion, upward assimilation” heuristic in group affiliation decisions. The present research also has implications for understanding how groups can strategically manage their reputation to attract prospective members, as well as how individuals make group-joining decisions at different stages of career development.

## 1. Introduction

Humans are inherently social beings, with their survival, development, and happiness dependent on group belonging (Begen & Turner-Cobb, 2015; Haslam et al., 2020; Hayward & Elliott, 2009; McNamara et al., 2021). In contemporary society, beyond the ascribed group memberships conferred by birth, individuals increasingly choose to join instrumental groups autonomously, such as professional associations, academic teams, and relational networks. This process of affiliation plays a crucial role in acquiring social resources, expanding relationship networks, and shaping social identity (Amiot et al., 2015; Robbins & Judge, 2022). Joining new groups typically means integrating group identity into one’s self-concept, so individuals need to carefully evaluate group characteristics. Among these evaluative dimensions, group competence—the perceived ability of a group to effectively execute tasks and achieve important outcomes—has emerged as a prominent factor influencing group selection (Amiot et al., 2015; Kachanoff et al., 2020; Wagoner & Hogg, 2016).

Group competence is a core dimension influencing group impression evaluation and has long been a central topic in social psychology research (Bettencourt et al., 2001; Dang & Liu, 2022; Fiske et al., 2002; Judd et al., 2005). In the stereotype content model, competence and warmth constitute the two fundamental axes by which individuals evaluate social objects, jointly shaping attitudes and evaluations toward groups (Brambilla & Leach, 2014; Nicolas et al., 2022). Among these, the competence dimension reflects judgments about a group’s ability to achieve targets, judgments typically based on external cues such as organizational status, resource control, and social influence (Cuddy et al., 2008). Compared to the warmth dimension, which is more susceptible to contextual influences, competence evaluations are typically more stable and structurally consistent (Nicolas et al., 2022; Fiske et al., 2016), thus becoming a key criterion in this study’s exploration of group affiliation decisions.

Previous research has generally regarded group competence as a positive attribute, suggesting that competent groups tend to receive more favorable evaluations. Fiske et al. (2007) proposed that people or groups perceived as both warm and competent are likely to elicit generally positive emotions and behavioral tendencies. In stereotype content research, competence is also closely associated with perceived status (Leach et al., 2007). Similarly, Brambilla et al. (2010) found that perceived group status positively predicts competence stereotypes. Moreover, obtaining status is an important motivation for individuals to join groups (Robbins & Judge, 2022). Therefore, we hypothesize that group competence has a positive influence on individuals’ willingness to join.

**Hypothesis 1** **(H1).** *Group competence positively influences individuals’ willingness to join.*

According to Social Identity Theory, individuals often define themselves through their group memberships rather than as isolated entities (Sato, 2025). To maintain a positive self-concept, individuals gravitate toward groups that are competent, high-status, and socially respected, as these affiliations directly enhance their social identity and self-esteem (Derks et al., 2009; Pool et al., 1998; Smith & Tyler, 1997; Stets & Harrod, 2004).

To operationalize individual competence, we drew on the SCM’s complementary analysis of individuals. When evaluating individuals, the Stereotype Content Model (SCM) similarly grounds social judgments along two fundamental dimensions: warmth and competence (Fiske, 2018; Fiske et al., 2002). The competence dimension typically encompasses attributes such as power, professional knowledge, and the capacity to achieve success (Abele et al., 2016). Within the SCM framework, competence is broadly defined as an individual’s or group’s capability and assertiveness and is closely linked to perceived social status. Importantly, the competence dimension is not unidimensional; it can be further decomposed into distinct sub-components, among which intelligence and talent have been identified as key constituents (Walzer & Czopp, 2011). Given that intelligence is conceptually and empirically closely related to power and professional knowledge, it provides a theoretically grounded basis for operationalizing competence—that is, perceived intelligence can serve as an effective experimental manipulation of the competence dimension.

Although high-competence groups may provide greater social identity value and external prestige, their attractiveness may depend on individuals’ own competence levels. Given a certain level of individual competence, joining a group with a higher average competence necessarily lowers the individual’s relative standing within that group. When individuals perceive their competence as significantly lower than the group level, they may experience feelings of marginalization or inadequacy, threatening identity-related needs and lowering self-esteem (Jetten et al., 2002). In such situations, individuals may develop negative self-evaluations and affiliation insecurity, potentially even leading to gradual disengagement from the group (Latrofa et al., 2012). Therefore, in real-world contexts, when individuals make group affiliation decisions, they consider not only the group’s competence (such as social status and resource acquisition) but also their relative status within the group and the resulting psychological experience (Smith & Tyler, 1997; Branscombe et al., 2002; Sacco & Bernstein, 2015). Consequently, we hypothesize that an individual’s willingness to join will be moderated by their own competence, with willingness decreasing when their competence is substantially lower than the group’s average competence level, as this may pose psychological threats (Derks et al., 2009; Jetten et al., 2002).

To further conceptualize the trade-off between individual competence and group competence, we introduced Optimal Distinctiveness Theory (Brewer, 1991; Leonardelli et al., 2010). According to Optimal Distinctiveness Theory, individuals strive to satisfy two fundamental and opposing social motives in group affiliation: the need for inclusion and the need for differentiation. When group competence is perceived as too exceptional, it may undermine individuals’ sense of uniqueness, thereby triggering identity threat and reducing affiliation motivation (Garcia et al., 2006). Conversely, groups that are only slightly more competent than the individual may provide an optimal balance between upward comparison and self-uniqueness, thus inspiring the strongest willingness to join (Klein & Kozlowski, 2000; Moon & Sung, 2015). Therefore, we hypothesize that individuals prefer groups that are slightly more competent than themselves. This preference reflects a trade-off: such groups simultaneously satisfy the need for inclusion (by not being too distant) and the need for differentiation (by providing a positive upward comparison).

Integrating the above theories and perspectives, we hypothesize that individual competence moderates the relationship between group competence and willingness to join. When group competence is lower than individual competence, since individuals always maintain an advantageous position within the group, their willingness to join is influenced only by group competence. The higher the group’s competence, the greater the social identity and external prestige individuals gain upon joining, thereby positively predicting individuals’ willingness to join. When group competence exceeds individual competence, individuals need to balance the high social identity brought by high-competence groups against their own lower status within the group. Simultaneously, according to Optimal Distinctiveness Theory, groups that are only slightly more competent than individuals are more attractive; therefore, as group competence increases, individuals’ willingness to join first rises and then falls.

**Hypothesis 2** **(H2).** *Individuals are most motivated to join groups that are slightly more competent than themselves, with their willingness to join decreasing as the competence gap widens further.*

This research explores the influence of group competence on individuals’ willingness to join. Based on the aforementioned theoretical foundation, we designed two empirical studies: Study 1 examines the direct effect of group competence on joining intention, and Study 2 introduces the variable of individual competence to investigate the role of individual-group matching in affiliation choices. Both studies were approved by the Ethics Committee of the School of Psychology, Central China Normal University, Wuhan, China (CCNU-IRB-202408019b), and the research protocols were preregistered on the Open Science Framework (OSF), accessed on 1 November 2024, with registration information as follows: https://os.psych.ac.cn/preregisterdetail/202410.00040.

Previous research has typically viewed group competence as dichotomous (Brambilla et al., 2011; e.g., “high” vs. “low”); however, this categorization approach may oversimplify the complexity of social judgment, leading to decreased statistical sensitivity and loss of subtle differences (Dixon et al., 2020; Streiner, 2002). To explore this issue more systematically, the present study categorized group competence and individual competence at different levels, examining in depth how individuals with varying competence levels are willing to join groups of different competence levels. This study operationalized group competence at five levels to simulate a continuous distribution, better reflecting the variability of competence in real-world group situations. This more nuanced manipulation provides a research framework with greater ecological validity and statistical robustness for examining how perceptions of group competence influence affiliation decisions.

## 2. Experiment 1

Experiment 1 manipulated group competence at five levels to examine individuals’ willingness to join groups of different competence levels.

We conducted a power analysis using G*Power 3.1 (Faul et al., 2009), assuming a medium effect size (Cohen’s f = 0.25) and setting the statistical power for repeated measures ANOVA at 0.80. The analysis indicated that a minimum sample size of 21 participants was required. The sample size in Study 1 exceeded this requirement.

### 2.1. Method

#### 2.1.1. Participants

A total of 50 participants were recruited from Credamo (https://www.credamo.com), an online research platform similar to MTurk. Participants were required to pass attention check questions embedded in the task to be included in the final analysis. Three participants failed the attention checks and were excluded. The final sample included 47 participants, of whom 36 were female. The mean age was 31.68 years (SD = 7.83). The study (including the experimental protocol and data collection) was conducted in accordance with the ethical principles for research involving human participants, as outlined in the World Medical Association Declaration of Helsinki.

#### 2.1.2. Experimental Design

Experiment 1 used a one-factor within-subjects design. The independent variable was group competence, which was manipulated at five levels: 15, 35, 55, 75, and 95. Participants evaluated all five groups. The dependent variables included participants’ willingness to join each group and their final group choice.

#### 2.1.3. Procedure

The study lasted approximately 15 min, and participants received 3 Chinese yuan as compensation. Data collection was conducted in September 2024. To maintain experimental control and avoid confounds introduced by specific task content, the nature of the test was deliberately left unspecified; participants were informed that the following scores represented the performance of different group members on a test. They were then asked to evaluate five groups with different competence levels based on their test scores: Group A (15 points), Group B (35 points), Group C (55 points), Group D (75 points), and Group E (95 points). The score distribution for each group was presented graphically, with the group average marked below the distribution curve. The study explained to participants: “The blue line represents the concentration area of group members’ scores, with the horizontal position of the curve’s peak corresponding to this score. The average score for each group is shown below the curve”.

**Figure 1:**
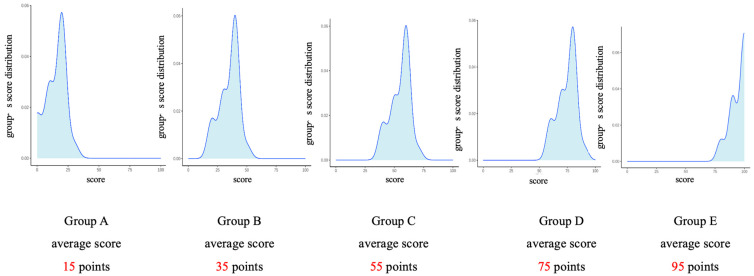
Presentation of group competence in Study 1.

Subsequently, participants reported their willingness to join each group and completed manipulation checks by evaluating the competence level of each group. At the same time, they were also required to select one group they most wanted to join. Finally, the study collected participants’ demographic information (such as gender and age) and provided an explanation and expressed gratitude to participants at the end of the experiment (Figure 1).

#### 2.1.4. Materials

Manipulation checks employed the competence dimension of the Stereotype Content Model in social cognition (Stereotype Content Model, Fiske et al., 2002; Wen et al., 2022). Participants rated each group on two competence-related adjectives—“competent” and “intelligent” (α = 0.70). The dependent variable was individuals’ willingness to join the group, measured through the item “I am very willing to join this group,” along with a forced-choice question: “Please select the group you most want to join.” All items used a 7-point Likert scale (1 = strongly disagree, 7 = strongly agree).

### 2.2. Result

#### 2.2.1. Manipulation Check

Before conducting the main analyses, we first verified the effectiveness of the competence manipulation. A repeated measures ANOVA on competence ratings revealed significant differences between groups, *F*(4, 184) = 113.58, *p* < 0.001, *η*^2^ = 0.71. The mean competence ratings increased as the assigned group competence level increased: 15-point group (*M* = 3.20, *SD* = 1.35), 35-point group (*M* = 3.54, *SD* = 1.33), 55-point group (*M* = 4.48, *SD* = 1.27), 75-point group (*M* = 5.60, *SD* = 0.76), and 95-point group (*M* = 6.43, *SD* = 0.47). Post hoc comparisons showed that most between-group differences were significant (*p*s < 0.001), except for the difference between the 15-point and 35-point groups, which did not reach significance (*p* = 0.11). These results indicate that the competence manipulation in this experiment was generally successful.

#### 2.2.2. Willingness to Join

**Figure 2:**
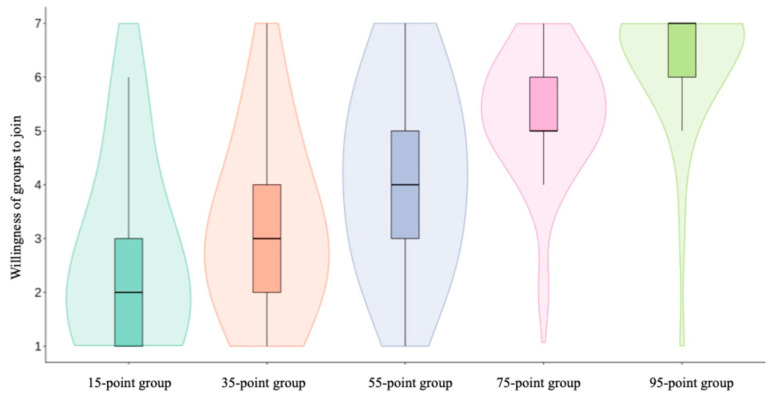
Willingness of groups to join in Experiment 1.

We examined the effect of group competence on individuals’ willingness to join (Figure 2). Results showed that group competence had a significant impact on joining willingness, *F*(4, 184) = 68.22, *p* < 0.001, *η*^2^ = 0.60. As group competence decreased, participants’ willingness to join gradually declined: 95-point group (*M* = 6.26, *SD* = 1.19), 75-point group (*M* = 5.34, *SD* = 1.13), 55-point group (*M* = 4.21, *SD* = 1.67), 35-point group (*M* = 3.19, *SD* = 1.53), and 15-point group (*M* = 2.62, *SD* = 1.65). Pairwise comparison results indicated that differences between all adjacent competence levels reached statistical significance (*p*s < 0.001).

In addition, gender was included as an independent variable to examine whether individuals’ willingness to join varied as a function of gender and group competence. The results indicated that the effect of gender was not significant (see Table 1).

#### 2.2.3. Group Selection

Participants’ final group choice was significantly influenced by the group’s objective competence, *χ*^2^(3) = 57.77, *p* < 0.001. Among the 47 participants, 34 chose Group E (72.34%), 7 chose Group D (14.89%), 5 chose Group C (10.64%), and 1 chose Group B (2.13%).

### 2.3. Discussion

This experiment aimed to investigate how group competence influences individuals’ willingness to join groups. By visually presenting group competence through distribution curves, we found significant differences in participants’ willingness to join five groups with different competence levels. Results indicate that the higher the group competence, the stronger participants’ willingness to join. This finding is further supported by participants’ actual selection behavior: 87.23% of participants chose high-competence groups (95-point group and 75-point group). These results confirm Hypothesis 1: individuals tend to select groups with stronger competence and higher social status positions.

## 3. Experiment 2

Experiment 2 aimed to investigate how individuals with different competence levels respond to groups with varying competence, and how these factors jointly influence willingness to join groups. In an online experiment, participants first completed a competence test containing 10 questions (selected from official civil service exam materials). After completing the test, participants were randomly assigned to one of three feedback score levels: 15 points, 55 points, or 95 points. These three levels represented low, medium, and high competence situations, corresponding to typical reference points in social comparison (i.e., worse than the group, equal to the group, better than the group). This simplified manipulation struck a balance between theoretical relevance and experimental feasibility. We used G*Power 3.1 (Faul et al., 2009) to calculate the sample size, assuming statistical power (*β*) of 0.80, medium effect size (Cohen’s f = 0.25), and repeated measures ANOVA as the analysis method, indicating that at least 78 participants were needed.

### 3.1. Method

#### 3.1.1. Participants

A total of 101 participants were recruited through Credamo (https://www.credamo.com), an online research platform similar to MTurk. Attention check questions and manipulation check items were included in the experiment. Fourteen participants failed either the attention checks or the manipulation checks and were excluded from the final analysis. The final sample consisted of 87 participants, including 27 females, with a mean age of 22.66 years (*SD* = 2.51). The study (including the experimental protocol and data collection) was conducted in accordance with the ethical principles for research involving human participants, as outlined in the World Medical Association Declaration of Helsinki.

#### 3.1.2. Experimental Design

Experiment 2 employed a 3 (individual competence level: 15, 55, 95) × 5 (group competence level: 15, 35, 55, 75, 95) mixed design, with individual competence as a between-subjects variable and group competence as a within-subjects variable. The dependent variables included participants’ willingness to join each group and their final group selection.

#### 3.1.3. Procedure

The procedure for Experiment 2 was basically consistent with Experiment 1, but with one key difference: participants first completed 10 competence test questions (see Supplementary Material). Following the logic of Raven’s Progressive Matrices (Raven & Raven, 2003), the competence test consisted of 10 numerical and figural reasoning items selected from the Chinese Civil Service Examination. Based on their assigned feedback scores, participants were divided into three different groups (15 points, 55 points, or 95 points).

The experimental instructions were as follows:

“You will first answer 10 competence test questions. The maximum answering time for each question is 1 min (a countdown will be displayed at the top of the screen). After each answer, your score will be displayed”.

Subsequently, participants reported their willingness to join each group, completed manipulation checks on group competence evaluations, selected the group they most wanted to join, indicated whether the intelligence test feedback matched their usual competence level, and provided demographic information (gender and age). Participants received 5 Chinese yuan as compensation after completing the experiment. Data collection was conducted in September 2024.

#### 3.1.4. Materials

All measurements were consistent with Experiment 1. Participants were not informed that the feedback was randomly assigned. The effectiveness of this manipulation was verified through a post-experimental check, in which participants indicated whether the test feedback matched their perceived usual competence level.

### 3.2. Results

#### 3.2.1. Manipulation Check

The results of the repeated measures ANOVA on group competence evaluation showed a significant effect, *F*(4, 340) = 121.21, *p* < 0.001, *η*^2^ = 0.59. The mean competence ratings increased as the assigned group competence level increased: 15-point group (*M* = 3.33, *SD* = 1.60), 35-point group (*M* = 3.78, *SD* = 1.47), 55-point group (*M* = 4.66, *SD* = 1.34), 75-point group (*M* = 5.56, *SD* = 0.89), and 95-point group (*M* = 6.24, *SD* = 0.99). Post hoc comparisons showed that most between-group differences were significant (*p*s < 0.001). This result confirms the successful manipulation of group competence, with differences between groups conforming to the manipulation.

#### 3.2.2. Willingness to Join

The results of a repeated-measures ANOVA on the effects of individual competence and group competence on willingness to join showed a significant main effect of group competence, *F*(4, 332) = 65.58, *p* < 0.001, *η*^2^ = 0.44. Participants’ willingness to join changed significantly with group competence level, showing a clear increasing trend: 95-point group (*M* = 5.73, *SD* = 0.15), 75-point group (*M* = 5.27, *SD* = 0.12), 55-point group (*M* = 4.53, *SD* = 0.15), 35-point group (*M* = 3.56, *SD* = 0.16), and 15-point group (*M* = 3.14, *SD* = 0.19). Pairwise comparisons between all groups reached statistical significance (*p*s < 0.05).

The main effect of individual competence was significant, *F*(2, 83) = 4.90, *p* = 0.01, *η*^2^ = 0.11. The willingness to join across different individual competence levels ranked from high to low was: 15-point individuals (*M* = 4.86), 95-point individuals (*M* = 4.28), and 55-point individuals (*M* = 4.19), with significant differences between all groups.

The interaction effect between group competence and individual competence was significant, *F*(8, 332) = 10.25, *p* < 0.001, *η*^2^ = 0.20. A simple effect analysis showed that for individuals with a competence score of 15 points, differences in group competence had no significant effect on willingness to join *F*(1, 83) = 1.90, *p* = 0.12. However, for individuals with competence scores of 55 points and 95 points, group competence significantly influenced willingness to join, reaching *F*(1, 83) = 635.91 and *F*(1, 83) = 687.17, *p*s < 0.001.

Post hoc test results showed that for individuals with a competence score of 55 points, the willingness to join the 95-point group (*M* = 5.54, *SD* = 1.50), 75-point group (*M* = 5.39, *SD* = 1.10), and 55-point group (*M* = 4.93, *SD* = 1.30) was significantly higher than the willingness to join the 35-point group (*M* = 3.00, *SD* = 1.41) and 15-point group (*M* = 2.11, *SD* = 1.47) (*p*s < 0.01), while the differences in willingness to join among the first three groups were not significant (*p*s > 0.05).

For individuals with a competence score of 95 points, their willingness to join showed a clear increasing trend: 95-point group (*M* = 6.34, *SD* = 0.90), 75-point group (*M* = 5.24, *SD* = 1.02), 55-point group (*M* = 3.93, *SD* = 1.46), 35-point group (*M* = 3.10, *SD* = 1.40), and 15-point group (*M* = 2.79, *SD* = 1.93). Except for the non-significant difference between the last two groups (*p* > 0.05), all other pairwise comparisons reached significant levels (*p*s < 0.001).

**Figure 3:**
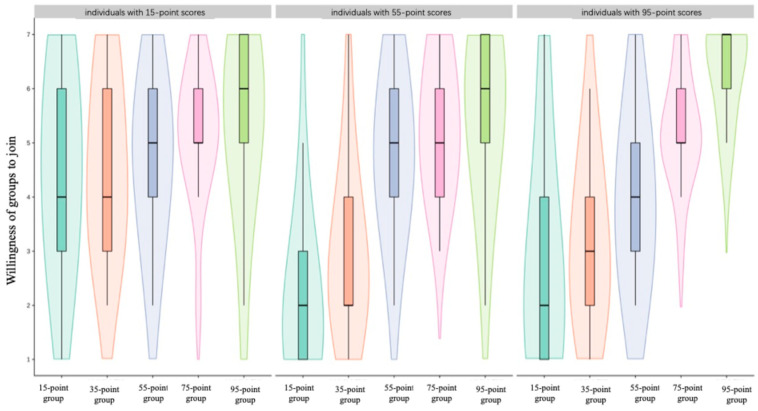
Willingness of groups to join in study 2.

In addition, gender was included as an independent variable to examine whether individuals’ willingness to join varied as a function of gender and group competence after accounting for individual competence. The results showed a significant interaction between gender and individual competence, whereas the other effects were not significant (see Table 2; Figure 3).

#### 3.2.3. Group Selection

Participants’ group choices were significantly influenced by group objective competence, *χ*^2^(4) = 73.29, *p* < 0.001. Among 87 participants, 48 chose to join Group E, 16 chose Group D, 12 chose Group C, 9 chose Group A, and 2 chose Group B.

For participants with personal scores of 15 points, *χ*^2^(4) = 10.83, *p* = 0.03, among 29 people, 31.03% chose Group E, 31.03% chose Group D, 6.90% chose Group C, 3.45% chose Group B, and 27.59% chose Group A.

For participants with personal scores of 55 points, *χ*^2^(4) = 17.72, *p* = 0.001, among 29 people, 41.38% chose Group E, 17.24% chose Group D, 34.48% chose Group C, 3.45% chose Group B, and 3.45% chose Group A.

For participants with personal scores of 95 points, *χ*^2^(1) = 21.55, *p* < 0.001, among 29 people, 93.10% chose Group E and 6.90% chose Group D.

### 3.3. Discussion

Study 2 examined the influence of group competence on individuals’ willingness to join under conditions of controlled individual competence. Results indicate that individual competence affects their tendency to join groups of different competence levels.

First, we found a main effect of group competence: individuals showed stronger preferences for high-competence groups, which is consistent with Hypothesis 1 and the findings of Study 1. Individuals tend to join high-competence groups primarily to gain positive social evaluation, affirm their self-worth, and enhance their social identity and self-esteem (Nakashima et al., 2013; Wolff et al., 2021).

Second, results revealed a main effect of individual competence. Individuals with low competence showed the strongest willingness to join, whereas those with medium competence showed the weakest willingness. Individuals with high competence also reported a relatively strong willingness to join.

Additionally, we found an interaction effect between individual competence and group competence. Low-competence individuals showed no significant difference in willingness to join when facing groups of different competence levels; high-competence individuals’ willingness to join varied significantly depending on group competence; medium-competence individuals showed no significant difference in willingness to join groups stronger than themselves but did show significant differences when facing groups of lower competence. Overall, individual competence shaped the pattern of willingness to join—when group competence was lower than one’s own, willingness to join increased as group competence increased; when group competence was higher than one’s own, willingness to join remained stable.

## 4. General Discussion

This study examined individuals’ willingness to join groups of different competence levels through two experiments. Experiment 1 explored how group competence affects willingness to join; Experiment 2 introduced individual competence, further investigating the interaction between self-competence and group competence.

Experiments 1 and 2 found that group competence positively predicts individuals’ willingness to join. This pattern is consistent with previous findings showing that affiliation with high-competence groups can enhance individuals’ positive self-image, social identity, and self-esteem (Robbins & Judge, 2022; Turner et al., 1979). In addition, individuals may experience social pressure during group evaluation and selection. To meet positive social expectations, they may be motivated to affiliate with high-competence groups (Asch, 1951). These findings suggest that individuals are generally motivated to choose groups that provide positive identity value and enhance their sense of self-worth.

In Experiment 2, individual competence was manipulated at three levels: high, medium, and low. The findings indicated that individual competence qualified the relationship between group competence and willingness to join. When group competence was lower than participants’ own competence, willingness to join increased with group competence. In contrast, when group competence exceeded participants’ own competence, willingness to join remained relatively stable. This suggests that individuals’ group-joining decisions depend not only on the absolute competence of the group but also on the relative competence relationship between the individual and the group.

This finding was only partially consistent with our original hypothesis. Based on Optimal Distinctiveness Theory (Leonardelli et al., 2010), we expected that individuals would prefer groups slightly more competent than themselves, because such groups may provide an optimal balance between inclusion and differentiation. We further expected that willingness to join would decline as the competence gap between the group and the individual became larger. However, the observed pattern did not fully support this prediction. When group competence was lower than participants’ own competence, willingness to join increased with group competence. By contrast, when group competence exceeded participants’ own competence, willingness to join remained relatively stable rather than decreasing. These findings suggest that the hypothesized inverted U-shaped relationship emerged only within the range in which group competence was lower than individual competence but was not observed when group competence exceeded individual competence.

One possible explanation for this inconsistency is that individuals may show downward aversion and upward homogenization in group-affiliation decisions. Specifically, individuals may use their own competence as a reference point when evaluating potential groups. When group competence is lower than their own, they may be particularly sensitive to competence differences and clearly distinguish between groups of different levels. Because lower-competence groups may provide limited identity value, status enhancement, and opportunities for self-improvement, individuals may be less willing to affiliate with them. Thus, as group competence approaches the individual’s own competence level, willingness to join increases.

However, when group competence exceeds individual competence, individuals may become less sensitive to further differences among higher-competence groups. That is, they may differentiate sharply among groups below their own competence level but perceive groups above their own competence level as similarly desirable. This pattern may reflect a process of upward homogenization, whereby individuals psychologically incorporate highly competent groups into their self-relevant ingroup schema. This interpretation aligns with the self-expansion model (Aron & Aron, 1986), which posits that individuals are motivated to include successful others within their self-concept to enhance their own sense of efficacy and worth. Rather than viewing highly competent groups as distant outgroups, individuals may regard them as potential or idealized ingroups. This cognitive adjustment may allow individuals to gain social identification and a sense of belonging while reducing the self-threat that can arise from competence gaps.

This explanation can also be understood in relation to Fei’s (1948/2008) “differential mode of association”, which emphasizes the flexible extension of self-related boundaries in social relations. In the present context, individuals may extend their psychological boundaries upward to include high-competence groups as desirable affiliation targets (Leach et al., 2008). In addition, from an evolutionary perspective, affiliation with high-competence groups may provide adaptive benefits, such as access to resources, learning opportunities, protection, and improved social status. These potential benefits may offset the discomfort associated with upward competence gaps.

Therefore, individuals may be reluctant to join groups below their own competence level, but once group competence exceeds their own, they may perceive these groups as similarly valuable and psychologically assimilate them into a desirable ingroup category. This may explain why willingness to join remained stable, rather than decreasing, as the competence gap widened among higher-competence groups.

Taken together, these findings suggest a novel “downward aversion, upward assimilation” heuristic in group affiliation decisions. When a group is perceived as less competent than the self, individuals are discriminating and avoidant; when a group is perceived as more competent, they become undiscriminating and accepting. This asymmetric pattern extends Optimal Distinctiveness Theory by revealing a boundary condition: the predicted need for differentiation appears operative primarily when individuals compare themselves to lower-competence groups but is overridden by assimilation motives when facing higher-competence groups.

## 5. Limitation

First, regarding the manipulation of competence, although we used a five-level group competence design to replace the traditional dichotomous design, successfully expanding the observational scope of the study and providing more nuanced experimental control, these discrete levels still have certain limitations. Specifically, this discrete division of competence levels may not fully capture how willingness to join changes progressively with subtle variations in group competence. In real-world situations, group competence typically presents as a continuous spectrum rather than distinctly separate levels. This discrete measurement method limits our precise characterization of the competence-willingness curve, especially potential non-linear relationships near critical points. Future research could consider using continuous measurement methods, such as sliding scales or more refined multi-point rating systems, to capture more subtle and dynamic relationship patterns between individuals’ perception of group competence and their willingness to join, thereby more accurately reflecting the subtle interactions between group competence and individual preferences and their possible threshold effects.

Second, regarding the manipulation of competence, the present study manipulated competence by varying intelligence scores. Although intelligence is an important component of competence, competence is a broader construct that may also include other aspects, such as social status, expertise, and achievement potential. Therefore, future research should further examine how different dimensions of competence influence individuals’ willingness to join groups. In addition, in Experiment 1, to avoid the influence of irrelevant variables, participants were only informed that the scores represented the performance of different groups on a test. Although the manipulation check confirmed the effectiveness of this manipulation, the relatively simple task context may have affected participants’ level of engagement. Future research should consider using more involved experimental tasks to enhance participants’ sense of participation and ecological validity.

Third, provided only partial support for the predictions derived from Optimal Distinctiveness Theory. When group competence exceeded individual competence, the reason why willingness to join remained relatively stable still requires further theoretical and empirical verification. Although the present study proposed “upward assimilation” as a potential explanation and interpreted this phenomenon through Social Identity Theory (Turner et al., 1979) and Fei’s (1948/2008) “differential mode of association,” this explanation still needs to be tested in future empirical research. In addition, several alternative explanations may also exist, such as demand characteristics, ceiling effects, or the influence of the task framing. Future studies should examine these possibilities empirically to provide a more comprehensive understanding of the psychological processes underlying this phenomenon.

Fourth, since we focused primarily on how individuals make initial joining decisions based on group competence, it may not fully capture the long-term development of social identity after formal membership is established. Social identity typically involves sustained perceptions of membership, the internalization of group norms, and a sense of belonging that develops through ongoing interaction. Future research should adopt longitudinal designs or field studies to examine whether competence alignment continues to predict group identity and sustained participation after individuals become formal group members, thereby providing a more comprehensive understanding of the role of group competence across different stages of group affiliation.

## 6. Conclusions

This study explores the relationship between group competence and individual willingness to join, finding that individuals’ willingness to join groups is positively influenced by the group’s competence level, while the relationship between group competence and joining willingness is moderated by individual competence. The findings partially supported Optimal Distinctiveness Theory. Specifically, when group competence is lower than individual competence, willingness to join increases as group competence improves toward the individual’s own level. However, when group competence exceeds that of the individual, willingness to join remains uniformly high rather than declining, exhibiting a phenomenon known as “upward homogenization”. This asymmetry suggests that the need for differentiation, central to Optimal Distinctiveness Theory, may be conditional on whether the comparison is downward or upward.

## Figures and Tables

**Table 1:** The results of repeated measures ANOVA on the effects of group competence and gender on willingness to join (experiment 1).

	*F*	*p*	*η* ^2^
Group competence	68.22	<0.001	0.60
Group competence × gender	1.13	0.34	0.01
Gender	0.76	0.39	0.00

**Table 2:** The results of repeated measures ANOVA on the effects of group competence, individual competence, and gender on willingness to join (experiment 2).

	*F*	*p*	*η* ^2^
group competence	65.58	<0.001	0.44
group competence × individual competence	10.25	<0.001	0.20
group competence × gender	0.36	0.84	0.00
group competence × individual competence × gender	0.22	0.99	0.00
individual competence	4.90	0.01	0.11
gender	0.22	0.64	0.00
individual competence × gender	4.83	0.01	0.03

## Data Availability

Due to privacy restrictions, data are not publicly available but can be provided by the corresponding author upon reasonable request.

## Supplementary Materials

The following supporting information can be downloaded at: https://www.mdpi.com/article/10.3390/bs16050821/s1, File S1: Competence test questions.
