# Supplementary material for: The Influence of Group Competence on Individual Willingness to Join
Source: Behav Sci (Basel). 2026 May 19;16(5):821. doi: 10.3390/bs16050821 (PMC13203655; doi:10.3390/bs16050821)
Supplement: Supplementary file 1 [file behavsci-16-00821-s001.zip › behavsci-4236407-supplementary.pdf]

File S1

$\frac{1}{59}$, $\frac{3}{70}$, $\frac{5}{92}$, $\frac{7}{136}$, ( )

A. $\frac{9}{272}$ B. $\frac{1}{224}$ C. $\frac{9}{224}$ D. $\frac{11}{224}$

4, $\frac{3}{2}$, $\frac{20}{27}$, $\frac{7}{16}$, $\frac{36}{125}$, ( )

A. $\frac{39}{144}$ B. $\frac{11}{54}$ C. $\frac{68}{196}$ D. 7

$-\sqrt{5}$，5，（　），25，$-25\sqrt{5}$

A．$-5\sqrt{5}$　　B．$5\sqrt{5}$　　C．$-15\sqrt{5}$　　D．$15\sqrt{5}$

100，20，2，$\frac{2}{15}$，$\frac{1}{150}$，（　）

A．$\frac{1}{3750}$　　B．$\frac{1}{225}$　　C．3　　D．$\frac{1}{500}$

1，4，13，40，121，(　)

A. 1093　　B. 364　　C. 927　　D. 264

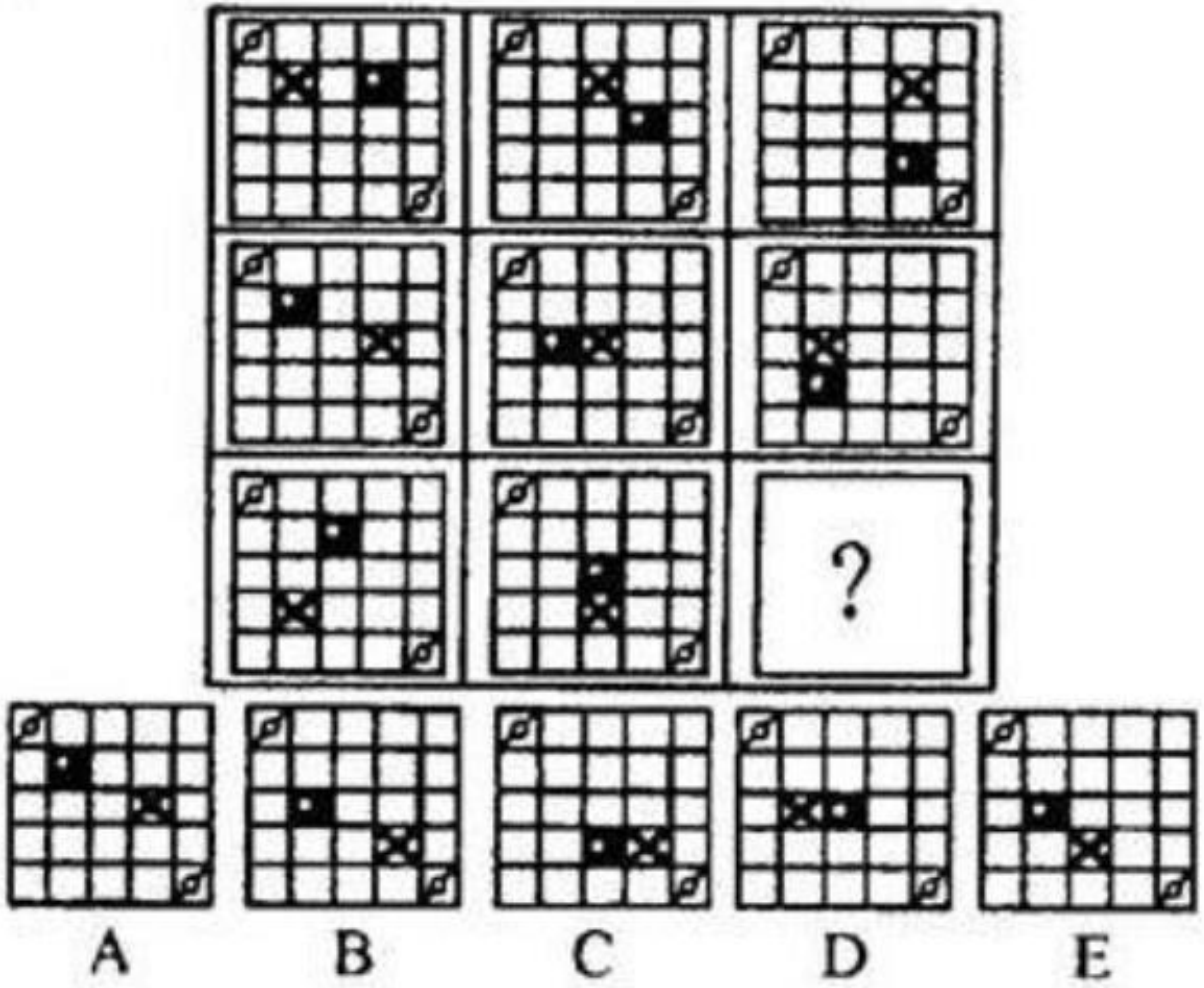
?
A
B
C
D
E

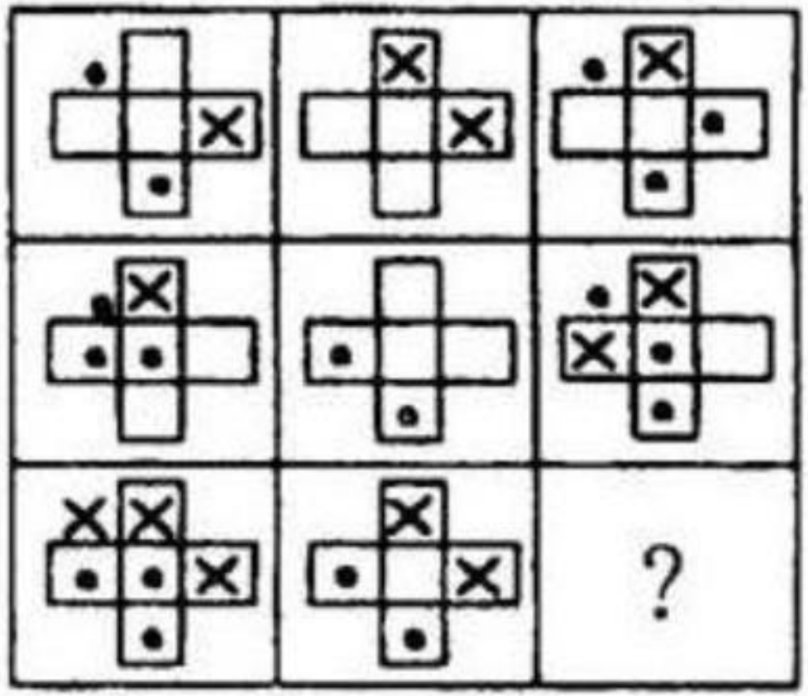

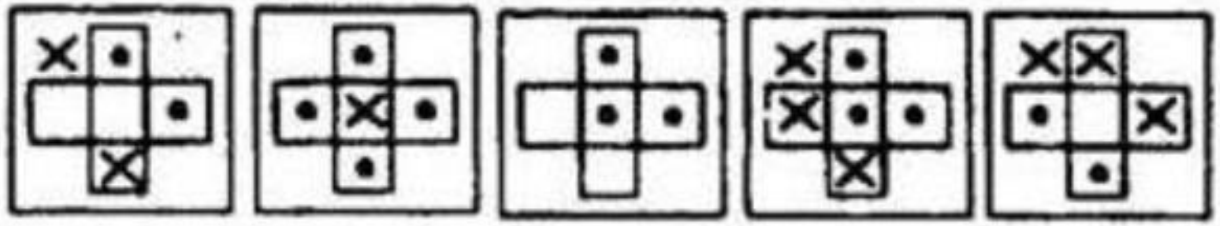

A B C D E

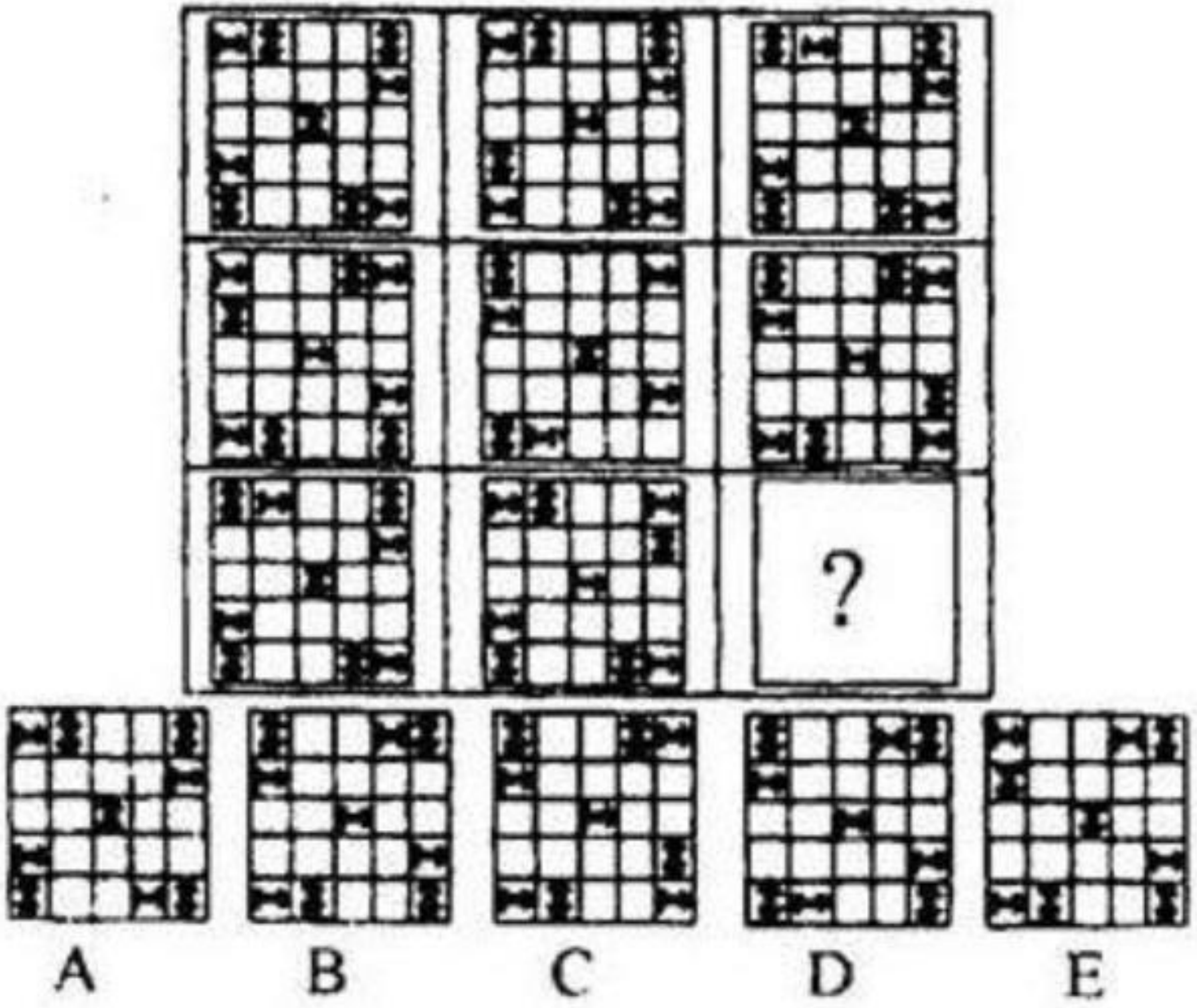
?
A
B
C
D
E

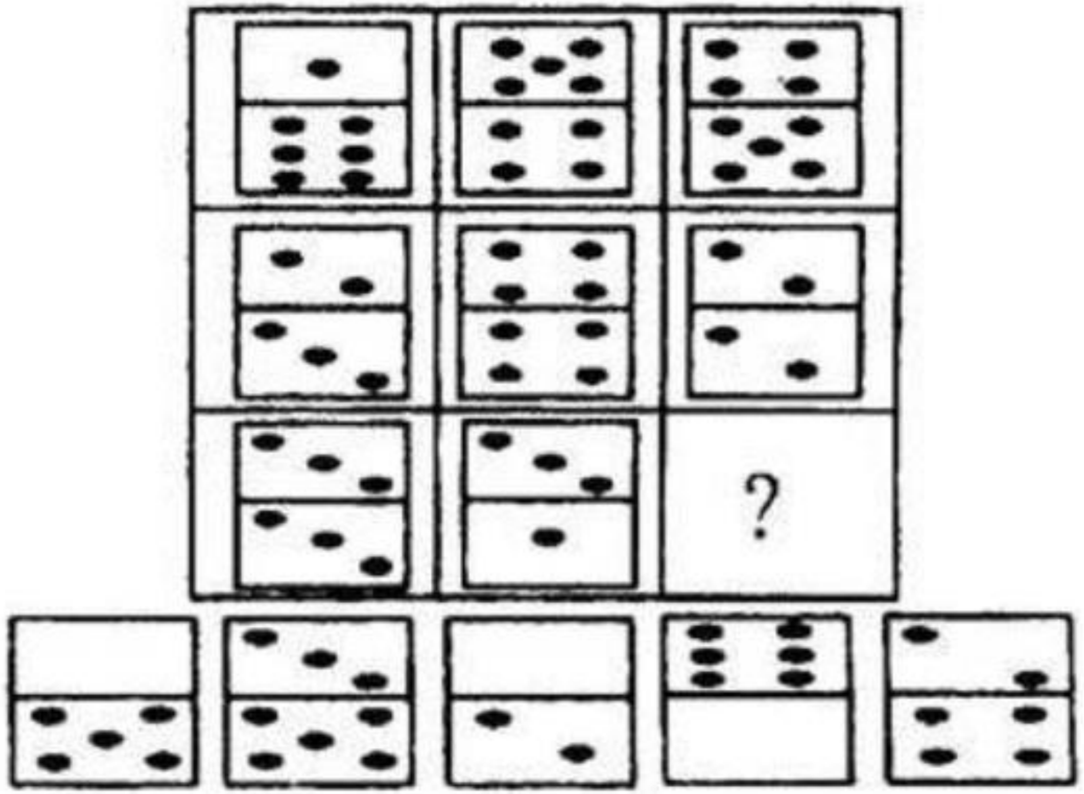

A B C D E

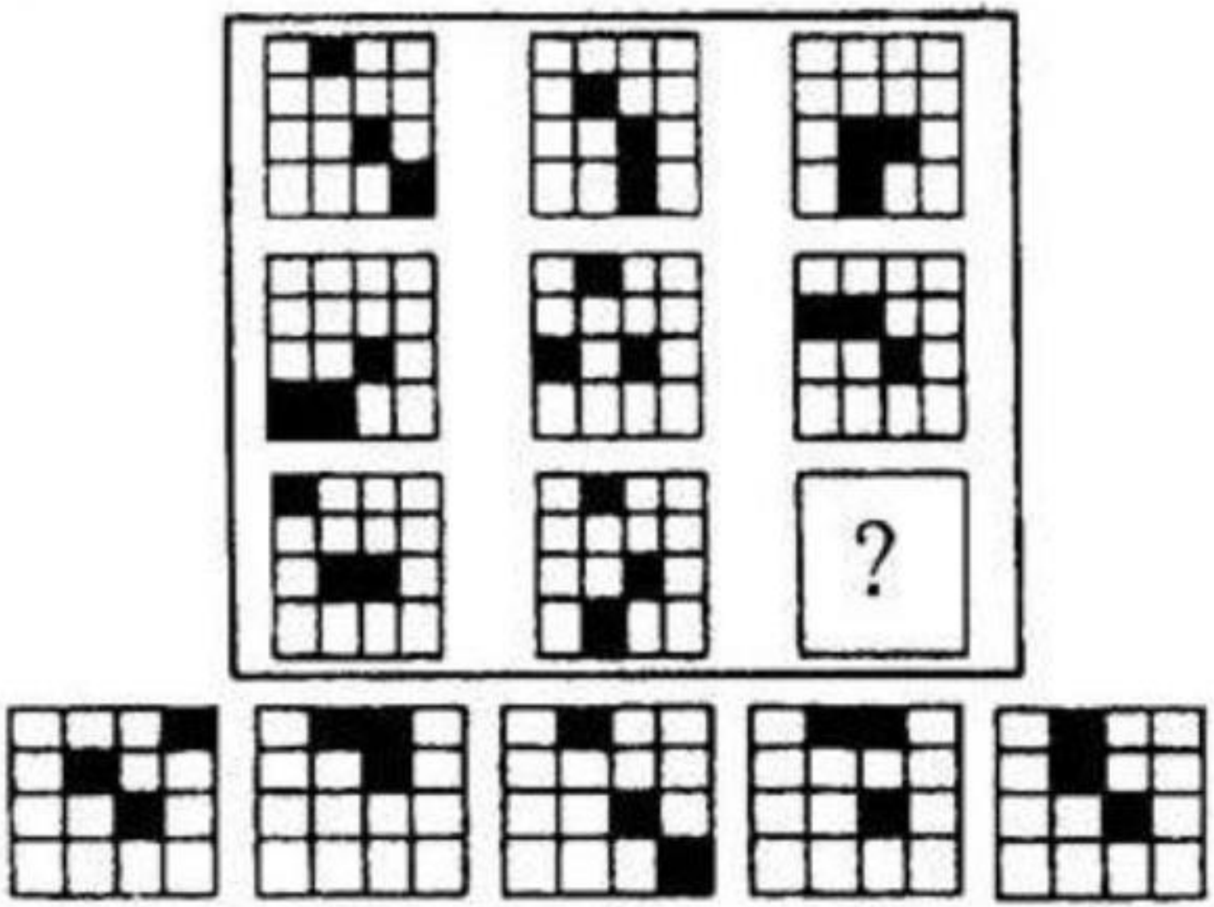

A B C D E
